# Aortic valve disease in diabetes: Molecular mechanisms and novel therapies

**DOI:** 10.1111/jcmm.16937

**Published:** 2021-09-24

**Authors:** Ileana Manduteanu, Dan Simionescu, Agneta Simionescu, Maya Simionescu

**Affiliations:** ^1^ Institute of Cellular Biology and Pathology “Nicolae Simionescu” of the Romanian Academy Bucharest Romania; ^2^ Department of Bioengineering Clemson University Clemson South Carolina USA

**Keywords:** aortic valve, calcification, diabetes, endothelial progenitor cells, high glucose, nanotherapy, stem cell therapy, tissue engineering, valvular endothelial cells, valvular interstitial cells

## Abstract

Valve disease and particularly calcific aortic valve disease (CAVD) and diabetes (DM) are progressive diseases constituting a global health burden for all aging societies (*Progress in Cardiovascular Diseases*. 2014;56(6):565: *Circulation Research*. 2021;128(9):1344). Compared to non‐diabetic individuals (*The Lancet*. 2008;371(9626):1800: *The American Journal of Cardiology*. 1983;51(3):403: *Journal of the American College of Cardiology*. 2017;69(12):1523), the diabetic patients have a significantly greater propensity for cardiovascular disorders and faster degeneration of implanted bioprosthetic aortic valves. Previously, using an original experimental model, the diabetic‐hyperlipemic hamsters, we have shown that the earliest alterations induced by these conditions occur at the level of the aortic valves and, with time these changes lead to calcifications and CAVD. However, there are no pharmacological treatments available to reverse or retard the progression of aortic valve disease in diabetes, despite the significant advances in the field. Therefore, it is critical to uncover the mechanisms of valve disease progression, find biomarkers for diagnosis and new targets for therapies. This review aims at presenting an update on the basic research in CAVD in the context of diabetes. We provide an insight into the accumulated data including our results on diabetes‐induced progressive cell and molecular alterations in the aortic valve, new potential biomarkers to assess the evolution and therapy of the disease, advancement in targeted nanotherapies, tissue engineering and the potential use of circulating endothelial progenitor cells in CAVD.

## 1 INTRODUCTION

Valve diseases and particularly calcific aortic valve disease (CAVD) and diabetes are progressive maladies and a global health burden for all aging societies.^1^, ^2^ Diabetic patients have a significantly greater propensity for cardiovascular disorders compared to non‐diabetic individuals.^3^, ^4^, ^5^ Accelerated CAVD is predictive of poor prognosis in valve disease and of faster degeneration of implanted bioprosthetic aortic valves.^6^ Patients with diabetes mellitus (DM) not only have an amplified risk of CAVD but also a significantly increased new incidence^7^, ^8^ of aortic stenosis, that progresses rapidly from mild to severe.^9^ Histopathological assessments showed a high degree of calcification in diseased aortic valves of DM patients compared to non‐diabetic patients.^10^ Clinical, histological and animal model experiments revealed the complexity of the processes leading to CAVD. Using an original experimental model of hyperlipidemia and hyperglycaemia, we have shown that the aortic valve was the first vascular territory greatly affected by these two aggressors.^11^ CAVD evolves progressively. It is a complex, cellular‐ driven process in which the valvular endothelial cells (VECs), valvular interstitial cells (VICs) and their interaction with the extracellular matrix have the key roles. VECs are the first cells to be affected by hyperglycaemia, whereas VICs are the key players in the process of valve calcification and mineralization.

To date, there are no pharmacological treatments available to reverse or retard the progression of CAVD. Traditional cardiovascular drugs like cholesterol‐lowering therapies (statins) and renin–angiotensin system blocking drugs have proven to be unsuccessful in slowing the progression of CAVD in clinical trials.^12^, ^13^, ^14^, ^15^ These findings suggest that despite the similarity of the risk factors that induce calcification of vascular and valvular structures, different mechanisms underlie the development and progression of their mineralization.^16^, ^17^, ^18^

Aortic stenosis (AS), the second most common indication for cardiac surgery, is most often treated by open‐heart or transcatheter aortic valve replacement^19^; both approaches are associated with a high risk of adverse events and substantial healthcare costs.^20^ Given that the burden of diabetes and CAVD will continue to increase worldwide in the coming decade and that currently, there is no reliable method of determining the optimal timing of intervention for a patient with asymptomatic AS or predicting when a patient will become symptomatic, a pharmacological method to reverse or slow the progression of CAVD is greatly needed. To this purpose, it is critical to continue to uncover the specific mechanisms of valve disease progression, to reveal accurate biomarkers for diagnosis and find new targets for therapies.

Despite significant advances in the field, to our knowledge, there are no specific targets or targeted therapies for the treatment of aortic valve disease in DM, urging for the need of new insights into the underlying cellular and molecular mechanism(s) on the basis of which new biomarkers, innovative bioengineering approaches and therapies for diabetes‐associated valve disease could be developed.

We present here an update on diabetes‐induced progressive changes in aortic valve structure and function, the advances in revealing molecular signatures and the new putative up‐regulated molecules that could be potential accurate biomarkers for diagnosis and/or targets for CAVD in diabetes. Novel therapeutic strategies, i.e. targeted nanotherapy addressed to the diseased cells, stem cell therapy and recent advancements in valvular tissue engineering are discussed. The recent advances on CAVD in diabetes bring confidence for the prospective progress in the treatment of this disorder and for the reduction of valve replacement surgery.

## 2 AORTIC VALVE STRUCTURE AND FUNCTION IS ALTERED IN DIABETES

### 2.1 Normal aortic valve structure and function

The aortic valve is a tricuspid valve responsible for maintaining the unidirectional blood flow from the left ventricle into the aorta. The valve consists of three thin semilunar cusps (thickness of less than 1 mm in humans) i.e. left, right and non‐coronary cusps, that are attached to a crown‐shaped annulus at the base.^21^ The thickness of the leaflets is not uniform but increases toward the free cusps margins.^22^ During the cardiac cycle, the cusps are exposed to various stresses, including pressure, tension and bending forces.^23^ The peak velocity of the blood flow through aortic valves during each cardiac systole is approximately 1.35 m/s,^24^ but in a calcified and thickened valve, the velocity may exceed 4 m/s, which eventually leads to progressive valvular stenosis. Biomechanical stimuli induce phenotypic and gene expression profile changes in valvular cells. The ventricular surface of the aortic cusps is exposed to unidirectional shear stress, while the aortic side is exposed to oscillatory shear stress^25^; as a consequence, the valvular endothelial cells (VECs) lining the two sides of the leaflets have distinct phenotypes and gene expression profiles.

Each leaflet has a trilaminar structure (fibrosa, spongiosa, and ventricularis), vital for the biomechanical properties of the aortic valve.^26^ The fibrosa, facing the aorta, is exposed to low shear stress secondary to diastolic, low velocity and disturbed blood flow. The fibrosa, containing mainly type I and type III collagen fibres, provides most of the tensile strength to the valve. The central layer, spongiosa, which represents about 60%–70% of the thickness of the cusp is rich in glycosaminoglycans (GAGs), components of proteoglycans that are highly hydrated, and act as “shock absorbers” during the cardiac cycle. The ventricularis, the layer facing the left ventricle, contains collagen and elastin fibres, providing more compliance; it grants the apposition of free edge leaflet regions and prevents the backward blood flow into the left ventricle during diastole.^27^, ^28^ The ventricular side of the cusps is exposed to high‐shear stress due to a systolic, high velocity and laminar blood flow. The normal valve leaflet is avascular^29^ but innervated by afferent and efferent nerves which contribute to valve function.^30^

As the entire cardiovascular system, the aortic valve surfaces are lined by endothelial cells that regulate vascular tone, inflammation, thrombosis and remodelling. They sense changes in shear stress and translate these mechanical stimuli into biological responses.^31^ There is evidence that VECs express von Willebrand factor, exhibit angiotensin converting enzyme activity and synthesize a rich extracellular matrix^32^; moreover, the cells are coupled by functional communicating (gap) junctions. VECs produce fibronectin, prostacyclin, hyaluronic acid and heparin‐like GAGs and are metabolically active taking up LDL.^33^, ^34^ Although VECs were shown to share many functions similar to endothelial cells (ECs) from other locations,^33^ they also display valve‐specific alignment, with a perpendicular orientation to flow, as compared to aortic ECs which respond to flow by aligning parallel to the direction of flow. Moreover, VECs response to shear stress was shown to be different when compared to aortic EC,^35^, ^36^ and the responses were found to be dependent on cytoskeletal reorientation. Interestingly, VECs display a different transcriptional profile compared to aortic EC.^31^

**FIGURE 1:**
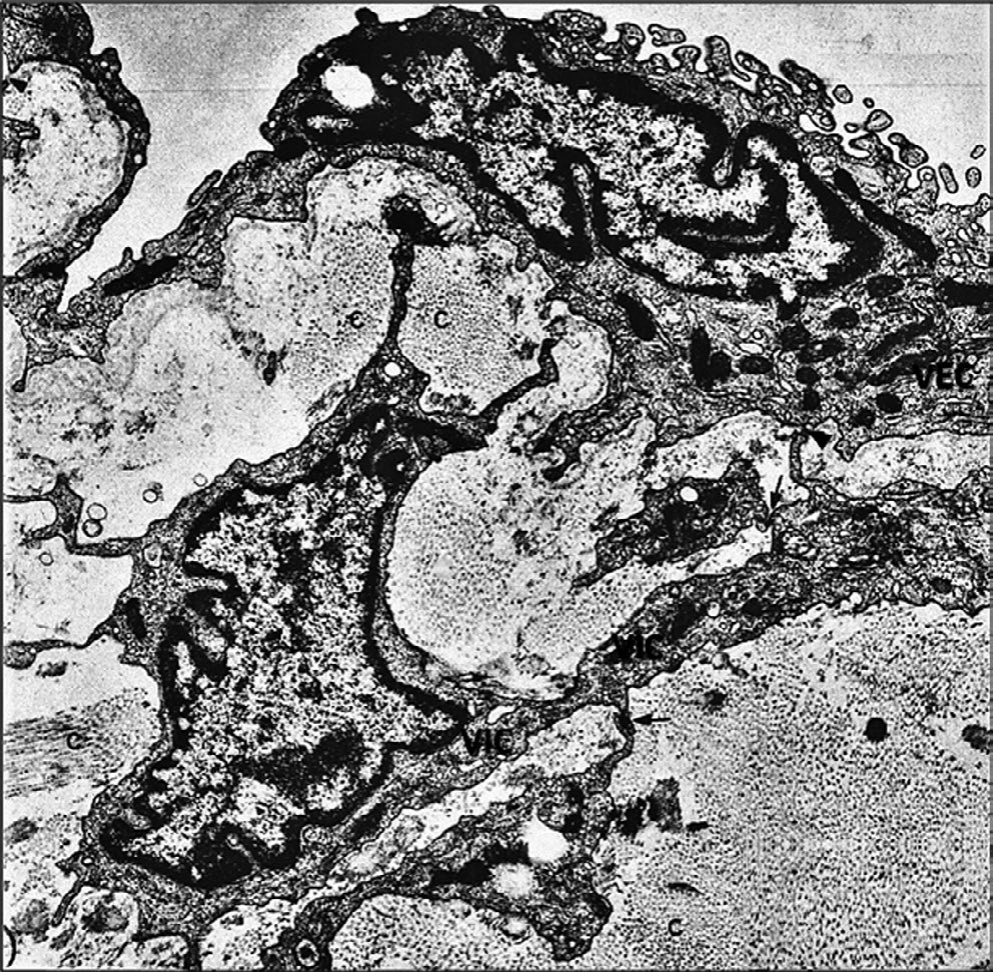
Electron micrograph depicting a fragment of a bicuspid valve (hamster). Valvular endothelial cells (VEC) with thick and thin segments line the ventricular side of the valve. Within the rich extracellular matrix, a valvular interstitial cell (VIC) lacking a basal lamina, exhibits numerous extensions that establish focal close apposition with VECs (arrowhead) and with neighboring VIC (arrows). x13,500. By permission from Circulation Research, 59, 3, 1986, p.13, Figure 2

The predominant cell population that resides in the valve interstitium are the valvular interstitial cells (VICs), which serve to maintain tissue homeostasis and structural integrity. As shown in Figure 1, these cells are embedded within the valve extracellular matrix (ECM), lack an organized basal lamina and present numerous slender extensions that establish contact with VECs and the neighbouring VICs.^37^ These cells are responsible for the generation, maintenance and repair of the ECM which is mainly composed of elastin, collagen and proteoglycans.^26^, ^38^ We have reported that VICs display both fibroblasts and vascular smooth muscle cells characteristics.^39^ Interactions between mechanical forces, valvular cells and the ECM influence remodelling potential and therefore durability of heart valves.

In addition to VICs, the valvular stroma is populated with some resident macrophages and very rare smooth muscle cells.^40^ A population of resident stem cells lying within the cusps has recently been identified.^41^ They appear to originate from the mobilization of hematopoietic‐derived stem cells towards cardiac valves,^42^ potentially contributing to normal or abnormal valve repair.

### 2.2 Diabetes‐induced changes in the aortic valve structure and function

**FIGURE 2:**
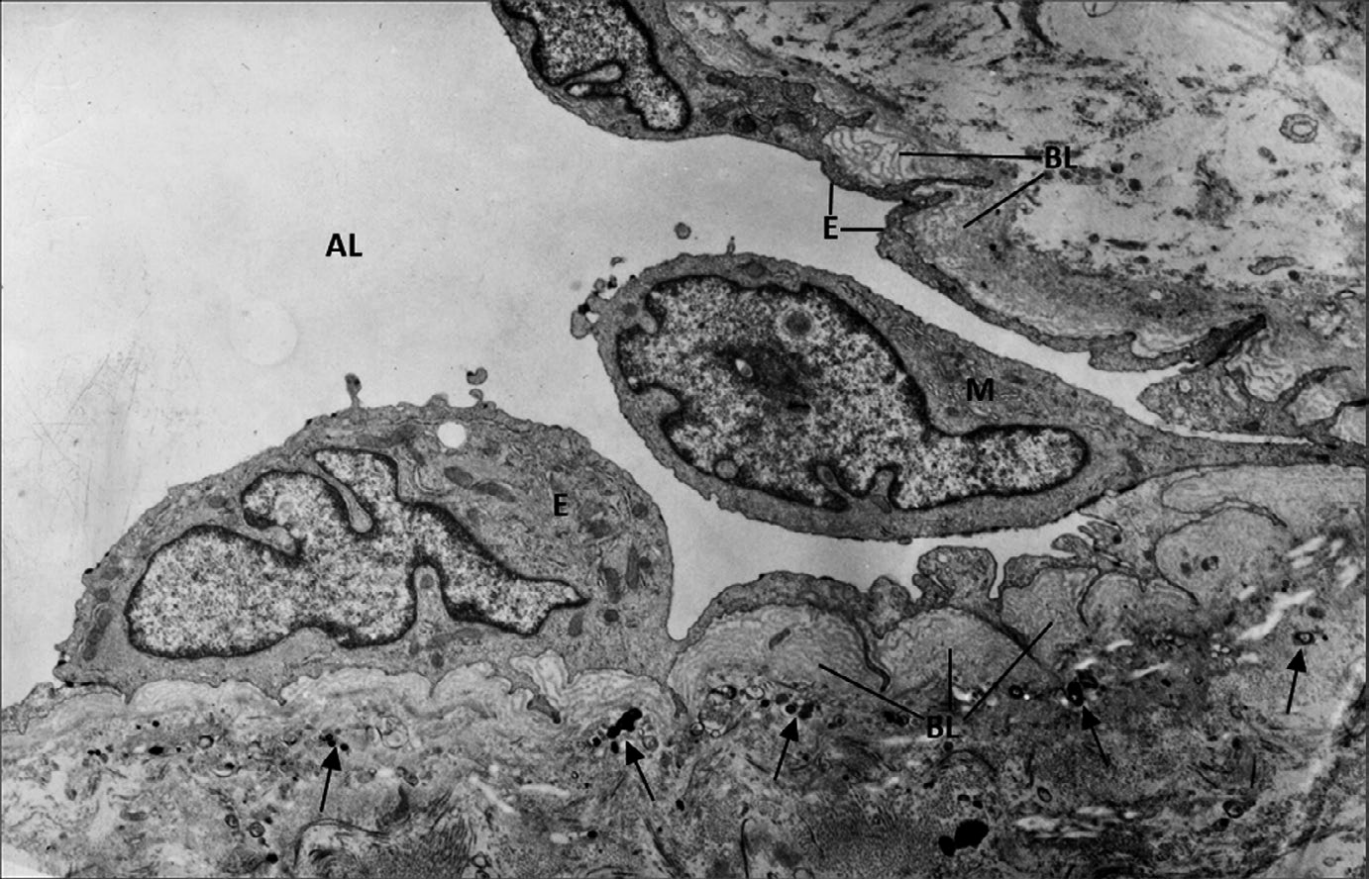
Early‐stage ultrastructural modifications of the aortic valve lesion occurred in a hyperlipemic/diabetic hamsters. Under a continuous endothelium (E) having thin areas intercalated within zones in which the cell is highly enriched in biosynthetic organelles, there is a characteristic hyperplasic, multilayered basal lamina (BL). The proliferated matrix contains numerous calcification cores (arrow). A plasma monocyte (M) insinuates a pseudopod between two valvular endothelial cells. (AL), aortic lumen. x7000. By permission from American Journal of Pathology, 148, 3, 1996, p. 1004, Figure 8

**FIGURE 3:**
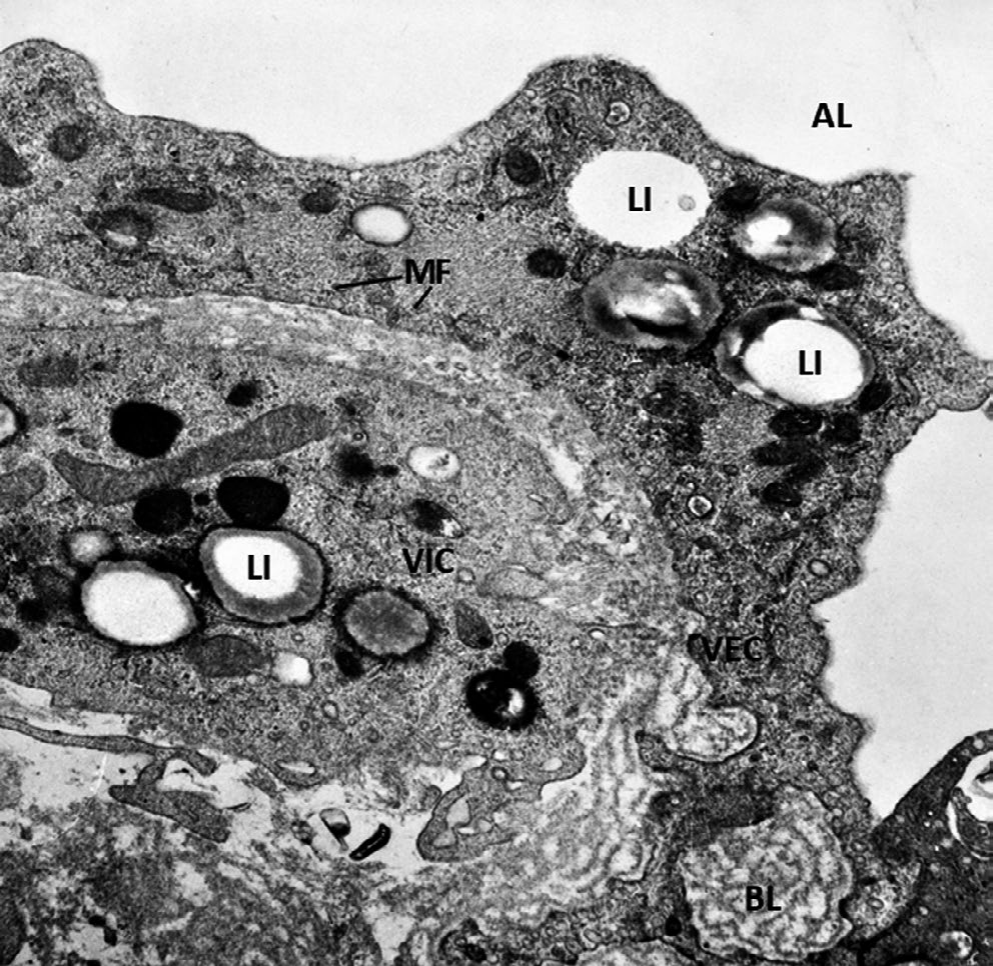
Ultrastructure of a lesion of the aortic valve in experimental hyperglycemia/hyperlipemia (4 weeks). The pathology progresses rapidly and the alterations include thickened VECs rich in organelles, microfilaments (MF), cytoplasmic lipid inclusions (LI), a multilayered basal lamina (BL) and the presence of valvular interstitial cell (VIC)‐ containing numerous lipid inclusions. X 24,000. By permission from American Journal of Pathology, 148, 3, 1996, p.1005, Figure 9

**FIGURE 4:**
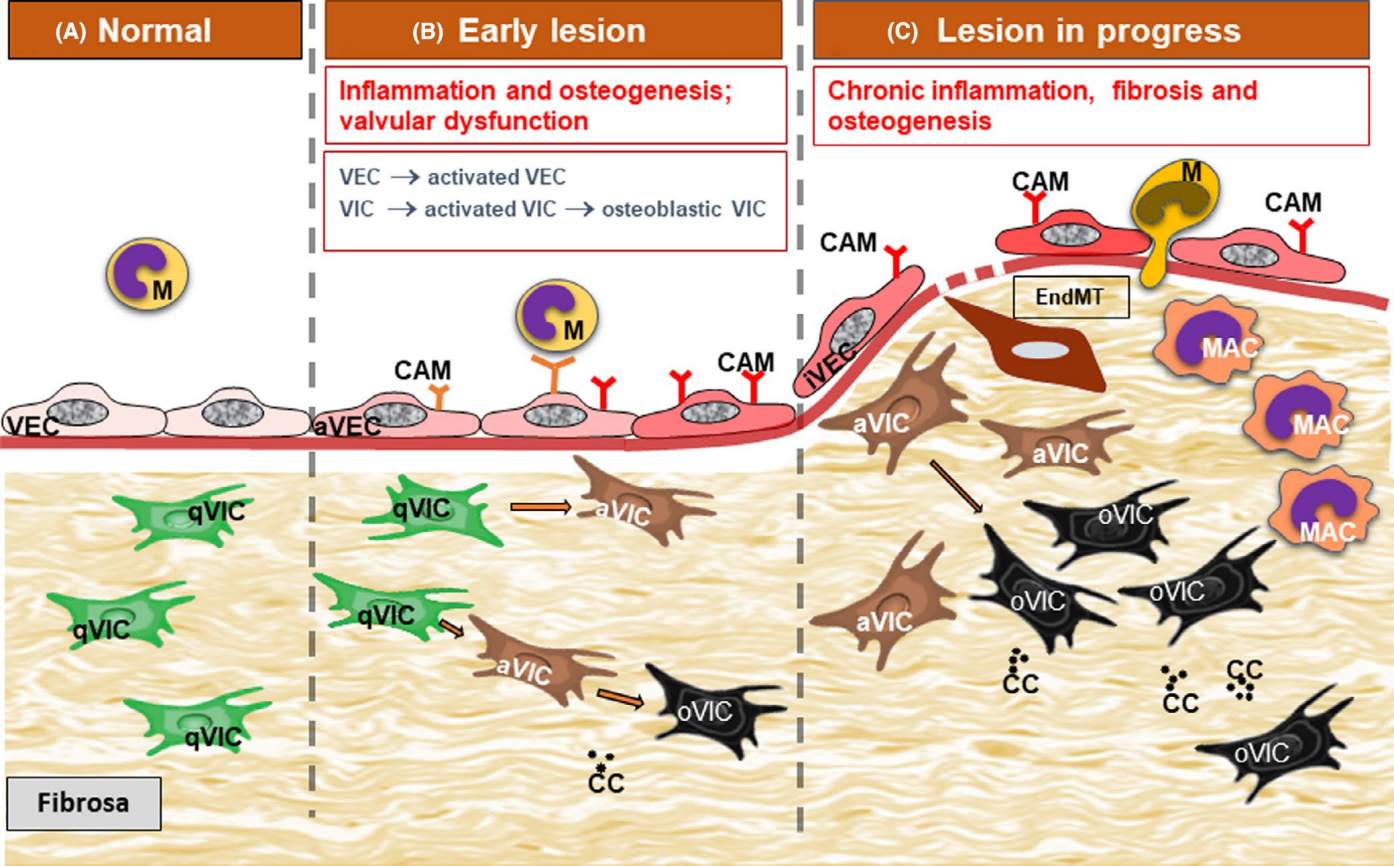
Diagram portraying diabetes‐induced accelerated progression of aortic valve atherosclerotic lesion. A. In normal conditions, valvular endothelial cells (VEC) and valvular interstitial cells (VIC) are quiescent ensuring valve homeostasis. B. The early lesion is characterized by the activation of normal VECs and the shift to a pro‐inflammatory phenotype (aVEC), the expression of new cell adhesion molecules (CAM) and subsequent monocyte (M) adhesion. VICs switch from the quiescent phenotype (qVIC) to an activated, myofibroblastic phenotype (aVIC) and an osteoblastic phenotype (oVIC). Moreover, calcification centers develop in the valvular stroma (CC). These alterations determine the onset of valvular dysfunction. C. With lesion progression, monocyte adhere and transmigrate through chronically inflamed VECs and switch to activated macrophages (MAC). Sometimes VECs undergo endothelial‐to‐mesenchymal transition (EndMT). There is an increased number of aVICs and oVICs and MAC in the valvular stroma. Chronic fibrosis develops and additional calcification centers appear, ultimately affecting valvular function

Experimental and clinical studies have shown that the aortic valve root is a lesion‐prone area for atherosclerotic plaque development one of the first and faster affected areas in animal models of combined diabetes and atherosclerosis.^11^ Notably, the changes were most consistently observed on the aortic aspect of the valve (fibrosa), the surface exposed to low shear forces and high hydrostatic pressures. In these areas, VECs acquire a synthetic phenotype and these changes are detected as early as 2 weeks after the onset of experimental diabetes. They are associated with a significant hyperplasia of the basal lamina which appeared in multiple interconnected layers in the meshes of which trapped oxidatively modified lipoproteins (initially called extracellular liposomes) were detected.^11^ These modified lipoproteins (mLp) were further demonstrated to be transcytosed from the plasma across VECs and accumulate within the subendothelial hyperplasic basal lamina.^43^ The VECs exposed on both sides to the hostile microenvironment, i.e. plasma hyperglycaemia/hyperlipidemia and subendothelial mLp, initiate a robust inflammatory reaction, displaying more and new cell adhesion molecules that induce adherence and diapedesis of blood monocytes (Figure 2). The activated VECs in hyperglycaemic‐hyperlipemic conditions also favour platelet adhesion.^44^ This is followed by the accumulation of lipid inclusions in VECs and in VICs and of lipid‐laden macrophages, the appearance of small calcification cores scattered throughout the valve ECM, all enclosed in an abundant collagen and microfibrils‐rich subendothelial matrix (Figure 3). With time, cholesterol crystals appear in VECs, VICs and in macrophage‐derived foam cells together with large calcification centres within an extensively proliferated stroma. All these changes occur at a much faster rate in experimental hyperlipidemia/hyperglycaemia than in experimental hyperlipidemia, alone.^11^ The onset and progression of the diabetes—accelerated aortic valve lesion is depicted in Figure 4.

In a mouse model of combined dyslipidemia and type 2 diabetes mellitus (the metabolic syndrome), the authors showed that within 6 months the model could reproduce pathophysiology of human AS, including inflammatory infiltrates, aortic valve fibrosis and upregulation of osteogenic genes, mineralization of the aortic leaflets, left ventricular dysfunction and the development of calcified aortic valve disease.^45^

Recently, we have reported that early diabetes induces aortic valve dysfunction in diabetic ApoE−/− mice fed with a hyperlipemic diet as detected by echography during the first week after the onset of diabetes. As importantly, we detected changes in the expression of molecules associated with inflammation, remodelling and osteogenesis. Remarkably, the peak aortic jet velocity (a marker of valve dysfunction) was highly correlated with an inflammatory biomarker (VCAM‐1), with pro‐osteogenic markers osteocalcin and alkaline phosphatase (ALP) with remodelling enzymes (MMP9) and with the myofibroblastic marker αSMA. These findings suggest that valvular dysfunction could develop even before clinical signs of AS and may be highly correlated with the specific molecular changes in valvular tissues. These highly expressed molecules may possibly become accurate biomarkers for diagnosis and targets for therapy.^46^

A comparison of the evolution of CAVD between diabetic and non‐diabetic subjects^10^ showed that the aortic valve leaflet macrocalcification was significantly enhanced in diabetic patients, whereas inflammation was similar in diabetic and non‐diabetic individuals. Moreover, Runx2 and ALP were detected to be significantly higher in diabetic patients suggesting that many valvular cells may undergo osteogenic differentiation. These studies, however, were restricted to surgically removed valve leaflets from patients with end‐stage disease and only highlight a snapshot in time of late‐stage events.^47^, ^48^ Future investigations involving comparative proteomics and transcriptomics analyses with larger sample sizes are warranted.

In another recent study,^49^ the role of small leucine‐rich proteoglycans in degenerative aortic valve disease and the influence of diabetes and hyperglycaemia on human aortic valves and valvular interstitial cells were examined. The results showed that biglycan, but not decorin or lumican was upregulated in degenerated human aortic valve cusps, hypothesizing that biglycan represents a potential link between degenerative aortic valve disease and diabetes.^49^

It was also shown that in patients diabetes is associated with increased valvular inflammation, measured by C‐reactive protein expression in patients’ valvular tissue.^50^ It was also demonstrated in diabetic patients that advanced glycation end products (AGEs) and AGE receptors (RAGE) accumulation is associated with AS severity; indeed, AGE‐related valvular collagen cross‐linking leads to enhanced inflammation, oxidative stress and calcification of the leaflets.^51^

Lately, a prospective cohort study has confirmed that DM is associated with an increased risk of AS.^52^, ^53^ However, other studies failed to demonstrate an association between AS progression and metabolic syndrome or diabetes during 3 years follow‐up; it was claimed that, in AS patients with well‐controlled DM, the effect of hyperglycaemia on AS severity is minor.^54^ More extensive clinical trials are needed to clarify this issue.

## 3 AORTIC VALVULAR CELLS PHENOTYPE IS PROGRESSIVELY MODIFIED IN DIABETES

### 3.1 In early diabetes valvular endothelial cells switch to a secretory and adhesive phenotype

As mentioned above, in a diabetic animal model, pathological changes occur particularly on VECs lining the aortic side of the valve, which is exposed to low shear forces and high hydrostatic pressure.^11^ Two weeks after onset of diabetes, VECs switched to a secretory phenotype, exhibiting an increased number of the rough endoplasmic reticulum elements, Golgi apparatus, and caveolae, features that correlate well with the progressive development of a multilayered basal lamina. Moreover, VECs exhibit a dramatic abundance of microfilaments, microtubules, centrioles and Weibel‐Palade bodies.^11^ This is followed by the VECs switch to an adhesive (pro‐inflammatory) phenotype, a process characterized by the expression of more and new surface adhesion molecules, that attract and induce adherence followed by diapedesis of blood monocytes (Figure 2). We have reported that short exposure of cultured VEC (24 h) to high glucose induces enhanced monocyte adhesion by mechanisms involving ICAM‐1, VCAM‐1, E‐selectin and CD18.^55^ Interestingly, the adhesivity of VEC for monocytes was higher than that of aortic ECs, results which may explain, in part, the propensity of cardiac valves for accelerated atherosclerosis in diabetes.^55^

### 3.2 Valvular endothelial cells undergo endothelial‐to‐mesenchymal transition

In diabetes in the aortic valve disease, an early event occurring in VECs in the aortic valve disease is the hyperglycaemia‐induced increased number of intermediary filaments and microtubules and attenuation of intercellular junctions.^11^ These features are associated with a switch of endothelial cells to a mesenchymal phenotype.

Endothelial‐to‐mesenchymal transition (EndMT) is a process by which the endothelial cells progressively acquire the phenotypic and functional characteristics of mesenchymal cells and express both endothelial and mesenchymal cell markers. In the heart valves development, EndMT is a physiological process. However, in the adult organisms when activated in the adult organisms, EndMT contributes to the progression of different diseases including diabetic nephropathy, diabetic renal fibrosis, cardiac fibrosis and atherosclerosis^51^, ^52^, ^53^, ^54^, ^55^, ^56^, ^57^, ^58^, ^59^ and has been shown to play a role in the pathogenesis of CAVD.^64^ Numerous in vivo and in vitro studies identified the EndMT‐related stimulants, such as inflammatory cytokines (TNF, IL‐6 and TGF), cellular transition features and underlying signalling pathways.^60^, ^61^

Although evidence of EndMT in VEC accumulated with time,^65^ the precise role of this process in the early‐ and end‐stage phases of CAVD is still unclear. Current data suggest that EndMT precedes osteogenic changes in VECs^61^ but more studies are needed to uncover the links between the process of EndMT and calcification.

Hyperglycaemia was shown to induce EndMT in several endothelia such as aortic EC, HUVECs, human retinal EC and glomerular EC. The process involves various mediators, for instance Ephrin B2, TGF‐β, angiotensin II, miR‐328 and signalling pathways, as FAK pathway, MAPK pathways and ROS/ERK1/2/MAPK‐dependent mechanisms.^62^, ^63^ Future, intensive investigations should focus on the role of these potential risk factors in mediating EndMT in CAVD.

### 3.3 Chronic hyperglycaemia induces an inflammatory phenotype of valvular endothelial cells

In search for the mechanism(s) of CAVD in diabetic environments, we developed a 3D model of human aortic valve leaflet, based on methacrylate gelatin populated with human VECs and VICs. The construct was exposed to chronic high glucose (HG) for 7 and 14 days, and the phenotypic changes of VECs and VICs were assessed.^64^, ^65^

**FIGURE 5:**
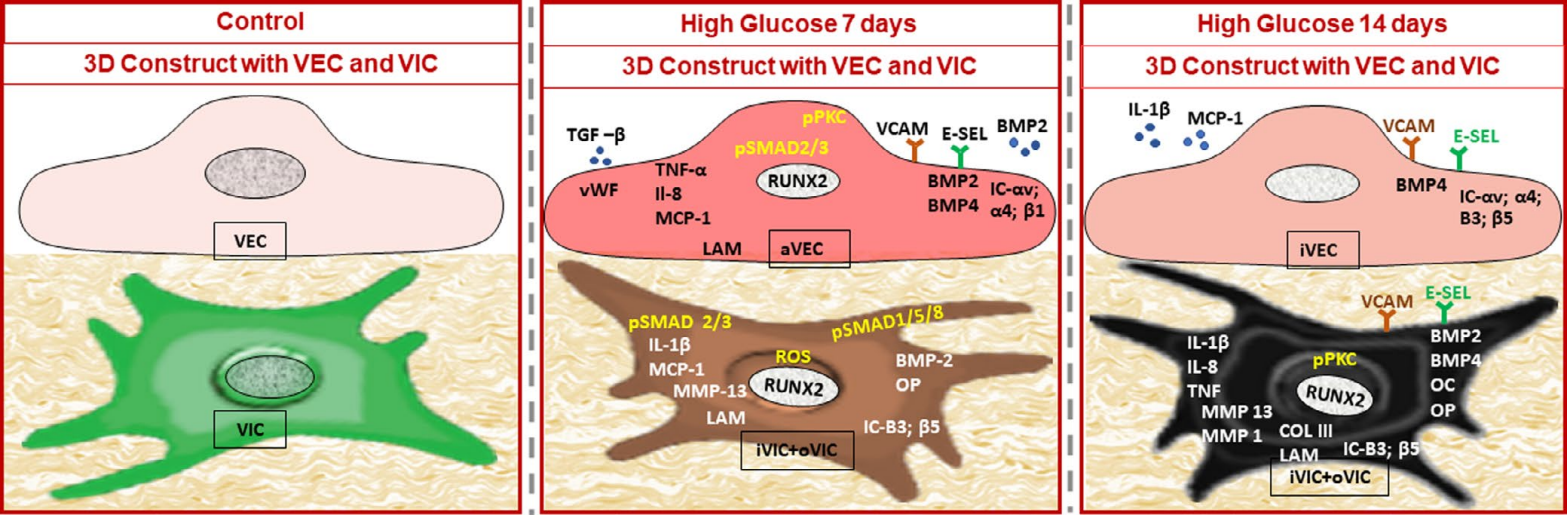
Diagram illustrating the high glucose (HG)‐induced progresive changes (at 7 and 14 days) of the expression of molecules in human VECs and VICs, as detected by an original 3D construct populated with valvular cells. *At 7 days*, the gene expression of von Willebrand factor (vWF), cytokines, cell adhesion molecules, integrins chains, laminin, pro‐osteogenic molecules: bone morphogenetic protein −2(BMP‐2), bone morphogenetic protein‐4 (BMP‐4) and RUNT‐related transcription factor 2 (RUNX2) is increased. SMAD2/3 and protein kinase K (PCK) proteins are activated. In VIC, note the enhanced expression of different cytokines, integrins, osteogenic molecules, and of RUNX2, matrix metalloproteinase 13 (MMP13) and laminin (LAM). HG activates SMAD1/5/8 and SMAD2/3 proteins and increases reactive oxygen species (ROS). Moreover, VEC and VIC in the 3D construct secrete in the conditioned media increased levels of transforming growth factor beta (TGF‐β) and bone morphogenetic protein‐2 (BMP‐2). *At 14 days*, in VEC, the gene expression of cell adhesion molecules, integrins and bone morphogenetic protein‐4 (BMP‐4) increases. Note that in VIC, HG increases the expression of numerous inflammatory molecules, cell adhesion molecules, pro‐osteogenic molecules: BMP‐2 and BMP‐4, matrix metalloproteinase (MMP), and of extracellular matrix protein collagen III (Col III). Phosphorylated protein kinase C (PKC) protein is activated. In addition, VEC and VIC in the 3D construct, in the conditioned media, secrete increased levels of interleukin‐1 β (Il‐1 β) and monocyte chemotactic protein‐1 (MCP‐1). Chronic HG induces in VEC mainly an inflammatory phenotype (aVEC), and in VIC, a mixed inflammatory (iVIC) and osteoblastic (phenotype (oVIC). Abbreviations: cytokines—tumor necrosis factor alpha (TNF‐α), interleukin 8 (Il‐8), cell adhesion molecules—vascular cell adhesion molecule 1 (VCAM‐1), e‐selectin (E‐sel), IC‐integrins chains, matrix metalloproteinase (MMP), laminin gamma chain (LAM), osteocalcin (OC), osteopontin (OP)

As shown in Figure 5, after 7 days of exposure to HG, VECs exhibit an increased expression of inflammatory molecules, cytokines and cell adhesion molecule. Moreover, VECs display an increase expression of BMP‐2, BMP‐4 and RUNX2. The canonical mediators of TGF‐beta signalling, SMAD 2/3 proteins and PKC‐alpha are activated by HG (increased phosphorylation). In addition, HG induces modifications of the interaction between VEC and ECM as suggested by the increased expression of the integrin chains, α4, αV and β1. Particularly, the increase in β1 integrin expression, the main integrin localized at the level of focal adhesions complexes interacting with collagen, suggests also changes in the pattern of focal adhesion complexes and their interaction with ECM.

After 14 days of cell exposure to HG, compared to controls, VEC exhibit an increased expression of cell adhesion molecules:VCAM‐1 and E‐selectin and of integrins αv, α4, β3 and β5.^65^ Interestingly, at this time, from the TGF‐beta family, only BMP‐4 expression is increased in VECs.^64^

Collectively, these data suggest that chronic HG induces in VEC mainly an inflammatory phenotype, but could have the capacity to adjust and control the expression of several pro‐inflammatory and pro‐osteogenic molecules. More studies will reveal the involvement of these cells in CAVD during the progression of diabetes.

### 3.4 Chronic hyperglycaemia induces an inflammatory and osteoblastic phenotype in valvular interstitial cells

As mentioned above, VICs, the predominant cell population found within the rich ECM of the aortic valve, are a heterogeneous population. Some VICs, like those found in porcine valves, exhibit a pericyte‐like behavior.^66^ Others, like VICs isolated from patients with CAVD, have angiogenic potential^67^ and in pathological conditions differentiate into osteoblast‐like cells and promote calcification.^39^, ^67^ Recently, using single‐cell RNA sequencing for the high‐throughput evaluation of heterogeneity in cells isolated from healthy human aortic valves, three subpopulations of VICs were defined by combining their developmental origin, localization, physical properties, morphology and molecular functions.^68^ VICs highly expressed the previously confirmed markers, collagen type IA1 (COL1A1) and type IIIA1 (COL3A1), substantiating that VICs are the primary resident cells in aortic valve tissues.

Activation of VICs is a normal regenerative process in the heart valve, but under pathological conditions, i.e. hyperlipidemia, diabetes or atherosclerosis, their activation leads to the inception of CAVD.^51^ There is evidence that elevated glucose levels, similar to those found in DM, enhance mineralization of cultured VICs.^69^

To uncover the mechanisms and the molecules implicated in the aortic valve calcification in diabetes, interesting data were obtained on VICs cultured in a 2D and 3D systems. Employing a 2D culture system, VICs did not show morphological changes and did not acquire an osteogenic phenotype in hyperglycaemia or hyperinsulinaemia.^70^ However, using a 3D model of human aortic valve, with VECs seeded on the surface of the construct previously encapsulated with VICs, we identified the inflammatory, remodelling and osteogenic changes induced by chronic HG in these cells.^64^, ^65^

As shown in Figure 5, VICs exposed for 7 days to HG exhibit an enhanced expression of pro‐inflammatory and osteogenic molecules. Importantly, HG increases the expression of RUNX2 transcription factor and activation of SMAD 2/3 and 1/5/8. In addition, HG increases the remodelling activity in VICs, as shown by the enhanced expression of MMP13 (Figure 5). Since HG induce also an increase in the level of reactive oxygen species (ROS), we assume that ROS could play an important role in the switch of VICs to an inflammatory and osteogenic phenotype. Together these results also demonstrate that a 3D valve model containing both VECs and VICs is closer to the in vivo conditions where communication between valvular cells is essential.

Compared to controls, after 14 days of HG exposure, VICs exhibit an increased gene expression of cytokines, cell adhesion molecules, integrins, remodelling and pro‐osteogenic molecules. In addition, the elevated level of the phosphorylated form of PKC‐α could be involved in the production of IL‐1β (Figure 5).

In summary, the above data indicate that chronic hg (that mimics diabetes conditions) induces in human vics co‐cultured with human vecs (in a 3d system), a concomitant inflammatory and osteoblastic phenotype and an increase in their remodelling activity. exposure of vecs and vics to osteogenic conditions leads to the development of calcium deposits, indicating that the 3d model is suitable to study valve calcification in diabetes.^64^

## 4 NEW THERAPEUTIC APPROACHES FOR AORTIC VALVE DISEASE IN DIABETES

Currently, there are no efficient pharmacological treatments to prevent or reverse CAVD. Lately potential medical approaches to circumvent this disease include nanotherapies, which are now beginning to be explored, stem cell therapies and more generally tissue engineering. Some of the advancement in these fields is briefly described below.

### 4.1 Nanotherapies

Interventions aiming to stop or reverse the osteoblastic transition of VIC may represent a therapeutic option for CAVD. A master transcription factor implicated in osteoblast differentiation is Runt‐related transcription factor 2 (Runx2) that regulates transcription and determines the increased expression of osteogenic genes such as collagen I, alkaline phosphatase (ALP), osteopontin (OSP), bone sialoprotein (BSP) and osteocalcin (OCN).^71^ Runx2 is not expressed in normal aortic valves, but its expression is induced in CAVD.^72^, ^73^, ^74^

An increased mRNA Runx2 level was determined in the aortic tissues of mice with combined dyslipidemia and type 2 diabetes compared to non‐diabetic and control mice.^45^ We have also detected an increased expression of Runx2 in VICs exposed to HG in a 3D model of human aortic valve.^64^ Thus, Runx2 is an important contributor to CAVD in diabetes/diabetic conditions.

Considering that the osteoblastic differentiation of VICs leads to aortic valve calcification and the role of Runx2 in the process, we have developed a nanotherapeutic strategy targeted to prevent the phenotipic differentiation of human aortic VICs into osteoblast‐like cells in diabetic and pro‐osteogenic conditions.^75^ We designed nanocarriers to silence Runx2, namely fullerene (C60)‐polyethyleneimine (PEI)/short hairpin (sh)RNA‐Runx2. We reported that these nanocarrriers efficiently downregulate Runx2 mRNA and protein expression leading subsequently to a significant reduction in the expression of osteogenic proteins (i.e. ALP, BSP, OSP and BMP4) in osteoblast‐committed VICs. These data indicated that silencing of Runx2 could represent a novel strategy to impede the osteoblastic phenotypic shift of VICs and the ensuing progress of CAVD. These results motivate further in vivo testing of this proof of concept. The efficient targeting of RUNX2, together with the advances in uncovering the significant molecules/pathways involved in VECs and VICs phenotypic alterations in diabetes, opens new avenues for developing innovative nanotherapeutics for the aortic valve disease in diabetes.

### 4.2 Stem cell therapy

In general, stem cell therapy was developed to correct the dysfunctional recruitment and homing of progenitor cells.^25^ The most studied sources of stem cells are the endothelial progenitor cells (EPCs) and the adipose‐derived stem cells (ADSCs).

EPCs represent a small fraction of the circulatory cells that are involved in vascular repair and angiogenesis. There are data that support the involvement of the αVβ3 and αVβ5 integrins in the adherence of EPC to denuded vessels.^76^ In patients with aortic stenosis, valvular endothelium regeneration is impaired not only by an increased senescence of VECs but also by a reduced number and function of circulating EPCs. It is generally accepted that diabetes reduces the number of EPCs and induces dysfunction in circulating EPCs, by mechanisms still uncovered.

In a recent study on streptozotocin‐induced diabetes in ApoE−/− mice, we showed that early‐stage diabetes superimposed on atherosclerosis generates alterations in EPC number, phenotype and homing.^76^ Importantly, in atherosclerosis‐prone mice, lower recruitment of EPCs in the aortic valve in early diabetes is the result of reduced EPC number and the decreased expression of α4β1 and αVβ3 integrins on EPCs; these results point to α4β1 and αVβ3 as new potential biomarkers and targets for therapy of the aortic valve in diabetes.^76^

It has been shown that PKA‐mediated phosphorylation of α 4β1integrin induced by high glucose plays a role in the bone marrow retention of EPCs.^77^ These results suggested two possible therapeutic interventions: a) bone marrow PKA inhibition that would help EPC mobilization, and b) administration of autologous or allogeneic EPCs modified to express higher levels of α4β1 and αVβ3 that will increase their adhesion at sites of vascular and valvular lesions.

Human ADSCs are another source of stem cell, often used in heart valve tissue engineering. Pluripotent mesenchymal stem cells found in relatively high number in the adipose tissue have the capacity to self‐renew and differentiate into many different types of cells; also, these cells synthesize collagen and elastin.^78^ Due to their relatively easy isolation and propagation in culture and their differentiation capacity, ADSCs are being employed now in preclinical studies. Currently, extracellular vehicles (EV) derived from ADSCs emerge as both diagnostic biomarkers and therapeutic tools in diabetes.^79^

### 4.3 Valvular tissue repair or regeneration employing biomaterials

Since heart valve tissues cannot regenerate spontaneously, replacement with artificial biological or mechanical heart valves, repair via reconstructive surgery or interventional catheterization is the current treatment option for management of advanced heart valve diseases.^25^ Artificial valves have a limited lifespan of ~10–15 years after implantation due to degeneration, calcification and thrombosis.^80^ Moreover, durability of artificial valves is further reduced to only 5–7 years in diabetic patients^81^ pointing to the need for further mechanistic studies. Recently, the group of Ferrari et al. have shown that oxidation and glycation contribute to the early demise of bioprosthetic heart valves in diabetic conditions.^82^ Tissue‐Engineered Heart Valves (TEHVs) might offer a new generation of cardiac valves aiming to overcome the limitations of the existing biological and mechanical heart valves^83^ Living TEHV could be capable of self‐regeneration and growth, with greater life span and better biocompatibility. Overall, TEHVs are still in their infancy period, and the translation to the clinic still faces many challenges.^25^ Notably, it is not known what the effect of diabetes would be on implanted TEHV, specifically on the scaffolds or directly on the cells. We have shown earlier that pre‐implantation treatment of the scaffolds with an antioxidant (penta galloyl glucose), a matrix binding polyphenol, reduces aortic valve scaffold biodegradation and calcification,^84^ thus opening avenues for development of TEHV resistant to diabetes.

## 5 FUTURE CHALLENGES AND OPPORTUNITIES

First believed that the aortic valve calcification is a passive degenerative process, it is now recognized that is a cell‐driven active process accomplished by resident cells, plasma‐recruited cells and the molecules they produce.

However, CAVD is still an enigma. To devise therapies (currently non‐existent), it is mandatory to uncover the molecular mechanisms underlying the implication of the valvular cells in the pathology of CAVD, in particular in diabetes, where hyperglycaemia deeply affects valvular cells and accelerates the disease.

The process is complex and multifactorial, involving, besides the valvular cells, cytokines, growth factors, matrix proteins, cell adhesion molecules, cytoskeletal components, transcription factors and signal transducers. A list of molecules exhibiting an enhanced gene and protein expression in the aortic valve in diabetes is shown in Table 1.

**TABLE 1:** Molecules exhibiting an enhanced gene and protein expression in the aortic valve in diabetes/diabetic conditions

Family	Name	Gene and proteins Increased expression	Cell Location	Condition	Model	Ref.
Cytokines	MCP−1	MCP−1 gene	VECs, VICs	HG	3D model of the human aortic valve	60
		Soluble MCP−1 protein	CM			
	TNF‐α	TNF‐α gene	VECs, VICs CM			
	IL8	IL8 gene				
	ILl‐β	ILl‐β gene				
	ILl‐β	Soluble ILl‐β protein				
Cell adhesion molecules	VCAM−1	VCAM−1 protein	VECs	HG	2D	50
		VCAM−1 gene	VECs, VICs	HG	3D	60
		VCAM−1 protein	Aortic valve	Early DM	HLD mouse	41
	ICAM−1	ICAM−1 protein				
	P‐selectin	P‐selectin protein				
	E‐selectin	E‐selectin protein	VECs	HG	2D	60
		E‐selectin gene	VECs, VICs	HG	3D	60
	IC α4	IC α4 gene	VECs			
	IC αv	IC αv gene				
	IC β1	IC β1 gene				
	IC β3	IC β3 gene	VECs, VICs			
	IC β5	IC β5 gene				
TGF‐β family members	TGF–β	Soluble TGF–β protein	CM	HG	3D	
		TGF–β protein	Aortic valve	Early DM	HLD mouse	41
	BMP−2	BMP−2 protein				
		Soluble BMP−2	CM	HG	3D	59
		BMP−2 gene	VECs, VICs			
	BMP−4	BMP−4 protein	Aortic valve	Early DM	HLD mouse	
		BMP−4 gene	VECs, VICs	HG	3D	60
Osteogenic molecules	OC	OC protein	Aortic valve	Early DM	HLD mouse	41
		OC gene	VICs	HG	3D	59
	OP	OP protein	Aortic valve	Early DM	HLD mouse	41
		OP protein		6‐mo DM	LAI mouse	40
		OP protein	VICs	βGP	2D	44
		OP gene		HG	3D	59
	ALP	ALP protein	Aortic valve	DM	DM patients	60
		ALP protein		Early DM	HLD mouse	
ECM proteins	FN	FN protein	Aortic valve	Early DM	HLD mouse	41
	LAM	LAM gene	VECs	HG	3D	60
	COL III	COL III gene	VICs			
MMPs	MMP−2	MMP−2 protein	Aortic valve	Early DM	HLD mouse	41
	MMP−9	MMP−9 protein				
	MMP−1	MMP−1gene	VICs	HG	3D	59
	MMP−13	MMP−13 gene				
SLPGs	BYG	BYG gene	Aortic valve	DM	DM patients	44
		BYG protein				
		BYG protein	VICs	βGP	2D	
AGEs	AGEs	AGEs	Aortic valve	DM	DM patients	46
			Plasma			
AGEs receptors	RAGE	RAGE	Human Aortic valve	DM	DM patients	46
			Plasma			
GPs	vWF	vWF gene	VECs	HG	3D	60
Actin protein family	α‐SMA	α‐SMA protein	Aortic valve	Early DM	HLD mouse	41
S100 family	S100‐A4	S100‐A4 protein	Aortic valve	Early DM	HLD mouse	41
Annexins	ANXII	ANXII protein	Aortic valve	DM	DM patients	5
TX factors	RUNX−2	RUNX−2 protein	Aortic valve	DM	DM patients	5
		RUNX−2 gene	VECs, VICs	HG	3D	
		RUNX−2 protein	VICs			59
Signal transducers	pSMAD1/5/8/9	pSMAD1/5/8/9 protein	VICs	HG	3D	59
	pSMAD 2/3	pSMAD 2/3protein	VECs, VICs			
	pPKC	pPKC protein				60

Abbreviations: 2D, Two‐dimensional model; 3D, Three‐dimensional model; AGEs, Advanced glycation end products; ALP, Alkaline phosphatase; ANX, Annexin; BMP, Bone morphogenetic protein; BYG, Biglycan; CM, Conditioned media; COL III, Type III collagen; DM, Diabetes mellitus; FN, Fibronectin; HG, High‐glucose media; HLD, Hyperlipemic diabetic; IC, Integrin chain; ICAM‐1, Intercellular adhesion molecule 1; IL‐1β, Interleukin 1 beta; IL‐8, Interleukin 8; LAI, Diabetes mellitus‐prone LDLr−/−/ApoB100/100/IGF‐II; LAM, Laminin gamma chain; MCP‐1, Monocyte chemoattractant protein1; MMP, Matrix metalloproteinase; OC, Osteocalcin; OP, Osteopontin; pPKC, Phosphorylated protein kinase C; pSMAD, Phosphorylated SMAD; RAGE, AGE receptor; Ref, Reference cited; RUNX‐2, Runt‐related transcription factor 2; S100‐A4, S100 calcium‐binding protein A4; SLPGs, Small leucine‐rich proteoglycans; TGF‐β, Transforming growth factor beta; TNF‐α, Tumor necrosis factor alpha; TX, Transcription; VCAM‐1, Vascular cell adhesion molecule 1; VECs, Valvular endothelial cells; VICs, Valvular interstitial cells; vWF, Von Willebrand factor; α‐SMA, α‐ smooth muscle actin; βGP, β‐Glycero‐phosphate stimulation.

The pioneering studies performed in experimental hyperlipidemia/hyperglycaemia by Dr Maya Simionescu's group identified the aortic valve as the first vascular territory affected by diabetes.^11^ Recently, employing murine models and in vitro studies using 3D valve scaffolds we have shown that early diabetes induces almost concurrently inflammatory and osteogenic modifications of the valvular interstitial cells leading to increased calcium deposition and overall valve dysfunction.^46^, ^64^, ^65^ Notably, our studies suggest that, in diabetic conditions, a significant inflammatory process occurs in the valve even in the absence of macrophage infiltration. Interestingly, the aortic valve dysfunction takes place at very early stages in diabetes, without detection of a statistically significant aortic valve thickening.

Recent studies focused on CAVD mechanisms led to the identification of new biomarkers for diagnosis and therapies. For example, Lipoprotein A was reported to correlate with the aortic valve calcification. Matrix‐remodelling‐associated protein 5 (MXRA5) and a fibronectin type III domain containing 1 (FNDC1), associated with ECM, were revealed as novel biomarkers of calcified valves.^85^ Lately, miRNAs were proposed as innovative biomarkers and therapeutic strategies for aortic valve stenosis, as revealed in a groundbreaking pre‐clinical study using inhibitors of miR‐34a.^86^, ^87^, ^88^

However, there are few data that identify biomarkers in CAVD in diabetes. Several differentially expressed “early” molecules which could serve as putative biomarkers for diagnosis and therapeutic targets in aortic valve disease in diabetes are presented in Table 1. Still, it remains a future challenge to identify accurate biomarkers to be employed for diagnosis and therapy for this disease.

Importantly, new nanocarriers aiming to block the shift of VICs to an osteoblastic phenotype were tested and found to be efficient in vitro; these results pave the way to the development of nanotherapies for CAVD.^75^ Another potential promise for treatment of CAVD is the recently emerged stem cell therapy. In addition, tissue engineering of AV attains continuously new improvements. A challenge for the future of valve replacement is to provide a viable valve populated with cells capable of self‐regeneration and growth, with greater life span, better biocompatibility and resistance to diabetes.^89^, ^90^, ^91^, ^92^

Meanwhile complex emerging technologies such as the next‐generation ‘omics’ techniques allows the study of the miRNAome, transcriptome, proteome and secretome^93^, ^94^, ^95^, ^96^; these big data will require analysis by improved bioinformatics tools which will lead to identification of the most relevant targets and the best therapeutic option. Transcriptional analyses of human aortic valves all focused on aortic stenosis were recently reported. Notably, a cell‐type transcriptome atlas of human aortic valves was elaborated showing the cell heterogeneity and the involvement of endothelial to mesenchymal transition in the evolution of calcific aortic valve disease.^73^ The study is an opportunity to understand the implication and the interaction of different valvular cells in CAVD in diabetes.

Another emerging perspective to be explored for the diagnosis and therapy of CAVD in diabetes are the extracellular vesicles (EV). There is evidence of the potential role of ADSC‐derived EVs in different pathologic conditions, either as biomarkers or as a direct effector or as a delivery system to target miRNAs to cells.^79^

Finally, to find relevant targets for therapy, new animal models^96^, ^97^ which could recapitulate the evolution of human AV disease need to be developed. Once obtained, the newly found therapeutical targets need to be further validated in 3D‐human aortic valve models,^64^, ^98^ as well as in large animals, for evaluation of safety and efficacy, before testing in clinical trials.^99^ These new data together with the present achievements in the biomedical science warrant a good perspective to find ways to prevent, slow‐down or reverse CAVD in diabetes.^100^

## CONFLICT OF INTEREST

The authors declare no conflict of interest.

## AUTHOR CONTRIBUTION

**Ileana Manduteanu:** Conceptualization (equal); Writing‐original draft (equal); Writing‐review & editing (equal). **Dan Simionescu:** Resources (equal); Visualization (equal); Writing‐review & editing (equal). **Agneta Simionescu:** Supervision (equal); Writing‐review & editing (equal). **Maya Simionescu:** Formal analysis (equal); Project administration (equal); Supervision (equal); Writing‐review & editing (equal).

## Data Availability

The dataset presented in this study is available from the corresponding author upon reasonable request.

## References

[1] Thaden JJ , Nkomo VT , Enriquez‐Sarano M . The global burden of aortic stenosis. Prog Cardiovasc Dis. 2014;56(6):565‐571. 10.1016/j.pcad.2014.02.006 24838132

[2] Driscoll K , Cruz AD , Butcher JT . Inflammatory and biomechanical drivers of endothelial‐interstitial interactions in calcific aortic valve disease. Circ Res. 2021;128(9):1344‐1370. 10.1161/CIRCRESAHA.121.318011. Epub 2021 Apr 29 PMID: 33914601. 33914601 PMC8519486

[3] Mazzone T , Chait A , Plutzky J . Cardiovascular disease risk in type 2 diabetes mellitus: insights from mechanistic studies. Lancet. 2008;371(9626):1800‐1809. 10.1016/S0140-6736(08)60768-0. PMID: 18502305; PMCID: PMC2774464. 18502305 PMC2774464

[4] Waller BF , Roberts WC . Cardiovascular disease in the very elderly. Analysis of 40 necropsy patients aged 90 years or over. Am J Cardiol. 1983;51(3):403‐421. 10.1016/s0002-9149(83)80072-1. PMID: 6823855. 6823855

[5] Yan AT , Koh M , Chan KK , et al. Association between cardiovascular risk factors and aortic stenosis: the CANHEART aortic stenosis study. J Am Coll Cardiol. 2017;69(12):1523‐1532. 10.1016/j.jacc.2017.01.025. PMID: 28335833. 28335833

[6] Lorusso R , Gelsomino S , Luca F , et al. Type 2 diabetes mellitus is associated with faster degeneration of bioprosthetic valve: results from a propensity score‐matched Italian multicenter study. Circulation. 2012;125:604‐614. 22203696 10.1161/CIRCULATIONAHA.111.025064

[7] Mazzone T , Chait A , Plutzky J . Cardiovascular disease risk in type 2 diabetes mellitus: insights from mechanistic studies. Lancet. 2008;371:1800‐1809. 18502305 10.1016/S0140-6736(08)60768-0 PMC2774464

[8] Katz R , Budoff MJ , Takasu J , et al. Relationship of metabolic syndrome with incident aortic valve calcium and aortic valve calcium progression: the Multi‐Ethnic Study of Atherosclerosis (MESA). Diabetes. 2009;58:813‐819. 19136658 10.2337/db08-1515 PMC2661576

[9] Banovic M , Athithan L , McCann GP . Aortic stenosis and diabetes mellitus: an ominous combination. Diab Vasc Dis Res. 2019;16:310‐323. 30623669 10.1177/1479164118820657

[10] Mosch J , Gleissner CA , Body S , Aikawa E . Histopathological assessment of calcification and inflammation of calcific aortic valves from patients with and without diabetes mellitus. Histol Histopathol. 2017;32:293‐306. 27353274 10.14670/HH-11-797 PMC5199639

[11] Simionescu M , Popov D , Sima A , et al. Pathobiochemistry of combined diabetes and atherosclerosis studied on a novel animal model. The hyperlipemic‐hyperglycemic hamster. Am J Pathol. 1996;148:997‐1014. 8774154 PMC1861738

[12] Rossebo AB , Pedersen TR , Allen C , et al. Design and baseline characteristics of the simvastatin and ezetimibe in aortic stenosis (SEAS) study. Am J Cardiol. 2007;99:970‐973. 17398194 10.1016/j.amjcard.2006.10.064

[13] Rossebo AB , Pedersen TR . Hyperlipidaemia and aortic valve disease. Curr Opin Lipidol. 2004;15:447‐451. 15243218 10.1097/01.mol.0000137229.00020.fe

[14] Teo KK , Corsi DJ , Tam JW , Dumesnil JG , Chan KL . Lipid lowering on progression of mild to moderate aortic stenosis: meta‐analysis of the randomized placebo‐controlled clinical trials on 2344 patients. Can J Cardiol. 2011;27:800‐808. 21742465 10.1016/j.cjca.2011.03.012

[15] Agmon Y , Khandheria BK , Meissner I , et al. Aortic valve sclerosis and aortic atherosclerosis: different manifestations of the same disease? insights from a population‐based study. J Am Coll Cardiol. 2001;38:827‐834. 11527641 10.1016/s0735-1097(01)01422-x

[16] van Rosendael PJ , Kamperidis V , Kong WK , et al. Comparison of quantity of calcific deposits by multidetector computed tomography in the aortic valve and coronary arteries. Am J Cardiol. 2016;118:1533‐1538. 27639685 10.1016/j.amjcard.2016.08.021

[17] Weisenberg D , Sahar Y , Sahar G , et al. Atherosclerosis of the aorta is common in patients with severe aortic stenosis: an intraoperative transesophageal echocardiographic study. J Thorac Cardiovasc Surg. 2005;130:29‐32. 15999037 10.1016/j.jtcvs.2004.11.040

[18] Vahanian A , Alfieri O , Andreotti F , et al. Joint Task Force on the Management of Valvular Heart Disease of the European Society of C and European Association for Cardio‐Thoracic S . Guidelines on the management of valvular heart disease (version. the joint task force on the management of valvular heart disease of the European society of cardiology (ESC) and the European association for cardio‐thoracic surgery (EACTS). Eur J Cardiothorac Surg. 2012;2012(42):S1‐S44. 10.1093/ejcts/ezs455 22922698

[19] Aronow WS . Indications for surgical aortic valve replacement. J Cardiovasc Dis Diagn. 2013;4(1):1‐4.

[20] Giritharan S , Cagampang F , Torrens C , Salhiyyah K , Duggan S , Ohri S . Aortic stenosis prognostication in patients with type 2 diabetes: protocol for testing and validation of a biomarker‐derived scoring system. JMIR Res Protoc. 2019;8:e13186. 31407670 10.2196/13186 PMC6818526

[21] IsmailEl‐HamamsyAdrian H , ChesterMagdi H . Yacoub Cellular regulation of the structure and function of aortic valves. Journal of Advanced Research. 2010;1(1):5‐12.

[22] Ho SY . Structure and anatomy of the aortic root. Eur J Echocardiogr. 2009;10:i3‐i10. 19131496 10.1093/ejechocard/jen243

[23] Chester AH , El‐Hamamsy I , Butcher JT , Latif N , Bertazzo S , Yacoub MH . The living aortic valve: from molecules to function. Glob Cardiol Sci Pract. 2014;2014(1):11. 10.5339/gcsp.2014.11 PMC4104380 25054122

[24] Balachandran K , Sucosky P , Yoganathan AP . Hemodynamics and mechanobiology of aortic valve inflammation and calcification. Int J Inflam. 2011;2011:263870. 21760982 10.4061/2011/263870 PMC3133012

[25] Taghizadeh B , Ghavami L , Derakhshankhah H , et al. Biomaterials in valvular heart diseases. Front Bioeng Biotechnol. 2020;8:529244. 33425862 10.3389/fbioe.2020.529244 PMC7793990

[26] Tseng H , Grande‐Allen KJ . Elastic fibers in the aortic valve spongiosa: a fresh perspective on its structure and role in overall tissue function. Acta Biomater. 2011;7:2101‐2108. 21255691 10.1016/j.actbio.2011.01.022 PMC4497587

[27] Lindman BR , Clavel MA , Mathieu P , et al. Calcific aortic stenosis. Nat Rev Dis Primers. 2016;2:16006. 27188578 10.1038/nrdp.2016.6 PMC5127286

[28] Mathieu P , Bouchareb R , Boulanger MC . Innate and adaptive immunity in calcific aortic valve disease. J Immunol Res. 2015;2015:851945. 26065007 10.1155/2015/851945 PMC4433691

[29] Alushi B , Curini L , Christopher MR , et al. Calcific aortic valve disease‐natural history and future therapeutic strategies. Front Pharmacol. 2020;11:685. 32477143 10.3389/fphar.2020.00685 PMC7237871

[30] Chester AH , Kershaw JD , Sarathchandra P , Yacoub MH . Localisation and function of nerves in the aortic root. J Mol Cell Cardiol. 2008;44:1045‐1052. 18485360 10.1016/j.yjmcc.2008.03.014

[31] White CR , Frangos JA . The shear stress of it all: the cell membrane and mechanochemical transduction. Philos Trans R Soc Lond B Biol Sci. 2007;362:1459‐1467. 17569643 10.1098/rstb.2007.2128 PMC2440408

[32] Manduteanu I , Popov D , Radu A , Simionescu M . Calf cardiac valvular endothelial cells in culture: production of glycosaminoglycans, prostacyclin and fibronectin. J Mol Cell Cardiol. 1988;20:103‐118. 2840511 10.1016/s0022-2828(88)80024-5

[33] Mongkoldhumrongkul N , Yacoub MH , Chester AH . Valve endothelial cells‐not just any old endothelial cells. Curr Vasc Pharmacol. 2016;14:146‐154. 26638797 10.2174/1570161114666151202205504

[34] Manduteanu IVE , Simionescu M . Uptake of LDL by valvular endothelial cells in culture. Rev Roum Biochim. 1990;27:235‐238.

[35] Butcher JT , Penrod AM , Garcia AJ , Nerem RM . Unique morphology and focal adhesion development of valvular endothelial cells in static and fluid flow environments. Arterioscler Thromb Vasc Biol. 2004;24:1429‐1434. 15117733 10.1161/01.ATV.0000130462.50769.5a

[36] Farivar RS , Cohn LH , Soltesz EG , Mihaljevic T , Rawn JD , Byrne JG . Transcriptional profiling and growth kinetics of endothelium reveals differences between cells derived from porcine aorta versus aortic valve. Eur J Cardiothorac Surg. 2003;24:527‐534. 14500070 10.1016/s1010-7940(03)00408-1

[37] Taylor PM , Batten P , Brand NJ , Thomas PS , Yacoub MH . The cardiac valve interstitial cell. Int J Biochem Cell Biol. 2003;35:113‐118. 12479860 10.1016/s1357-2725(02)00100-0

[38] Chen JH , Simmons CA . Cell‐matrix interactions in the pathobiology of calcific aortic valve disease: critical roles for matricellular, matricrine, and matrix mechanics cues. Circ Res. 2011;108:1510‐1524. 21659654 10.1161/CIRCRESAHA.110.234237

[39] Filip DA , Radu A , Simionescu M . Interstitial cells of the heart valves possess characteristics similar to smooth muscle cells. Circ Res. 1986;59:310‐320. 3769149 10.1161/01.res.59.3.310

[40] El‐Hamamsy I , Chester AH , Yacoub MH . Cellular regulation of the structure and function of aortic valves. J Adv Res. 2010;1(1):5‐12. 10.1016/j.jare.2010.02.007

[41] Chen JH , Yip CY , Sone ED , Simmons CA . Identification and characterization of aortic valve mesenchymal progenitor cells with robust osteogenic calcification potential. Am J Pathol. 2009;174:1109‐1119. 19218344 10.2353/ajpath.2009.080750 PMC2665769

[42] Visconti RP , Ebihara Y , LaRue AC , et al. An in vivo analysis of hematopoietic stem cell potential: hematopoietic origin of cardiac valve interstitial cells. Circ Res. 2006;98:690‐696. 16456103 10.1161/01.RES.0000207384.81818.d4

[43] Dobrian A , Mora R , Simionescu M , Simionescu N . In vitro formation of oxidatively‐modified and reassembled human low‐density lipoproteins: antioxidant effect of albumin. Biochim Biophys Acta. 1993;1169:12‐24. 8334145 10.1016/0005-2760(93)90076-l

[44] Manduteanu I , Calb M , Lupu C , Simionescu N , Simionescu M . Increased adhesion of human diabetic platelets to cultured valvular endothelial cells. J Submicrosc Cytol Pathol. 1992;24:539‐547. 1458440

[45] Le Quang K , Bouchareb R , Lachance D , et al. Early development of calcific aortic valve disease and left ventricular hypertrophy in a mouse model of combined dyslipidemia and type 2 diabetes mellitus. Arterioscler Thromb Vasc Biol. 2014;34:2283‐2291. 25231636 10.1161/ATVBAHA.114.304205

[46] Tucureanu MM , Filippi A , Alexandru N , et al. Diabetes‐induced early molecular and functional changes in aortic heart valves in a murine model of atherosclerosis. Diab Vasc Dis Res. 2019;16:562‐576. 31530180 10.1177/1479164119874469 PMC6787765

[47] Aikawa E , Otto CM . Look more closely at the valve: imaging calcific aortic valve disease. Circulation. 2012;125:9‐11. 22090164 10.1161/CIRCULATIONAHA.111.073452

[48] New SE , Aikawa E . Molecular imaging insights into early inflammatory stages of arterial and aortic valve calcification. Circ Res. 2011;108:1381‐1391. 21617135 10.1161/CIRCRESAHA.110.234146 PMC3139950

[49] Barth M , Selig JI , Klose S , et al. Degenerative aortic valve disease and diabetes: Implications for a link between proteoglycans and diabetic disorders in the aortic valve. Diab Vasc Dis Res. 2019;16:254‐269. 30563371 10.1177/1479164118817922

[50] Natorska J , Wypasek E , Grudzien G , et al. Does diabetes accelerate the progression of aortic stenosis through enhanced inflammatory response within aortic valves? Inflammation. 2012;35:834‐840. 21935671 10.1007/s10753-011-9384-7 PMC3332381

[51] Kopytek M , Zabczyk M , Mazur P , Undas A , Natorska J . Accumulation of advanced glycation end products (AGEs) is associated with the severity of aortic stenosis in patients with concomitant type 2 diabetes. Cardiovasc Diabetol. 2020;19:92. 32552684 10.1186/s12933-020-01068-7 PMC7301463

[52] Kamalesh M , Ng C , El Masry H , Eckert G , Sawada S . Does diabetes accelerate progression of calcific aortic stenosis? Eur J Echocardiogr. 2009;10:723‐725. 19406839 10.1093/ejechocard/jep048

[53] Larsson SC , Wallin A , Hakansson N , Stackelberg O , Back M , Wolk A . Type 1 and type 2 diabetes mellitus and incidence of seven cardiovascular diseases. Int J Cardiol. 2018;262:66‐70. 29605469 10.1016/j.ijcard.2018.03.099

[54] Testuz A , Nguyen V , Mathieu T , et al. Influence of metabolic syndrome and diabetes on progression of calcific aortic valve stenosis. Int J Cardiol. 2017;244:248‐253. 28684044 10.1016/j.ijcard.2017.06.104

[55] Manduteanu I , Voinea M , Serban G , Simionescu M . High glucose induces enhanced monocyte adhesion to valvular endothelial cells via a mechanism involving ICAM‐1, VCAM‐1 and CD18. Endothelium. 1999;6:315‐324. 10475094 10.3109/10623329909078498

[56] Kovacic JC , Dimmeler S , Harvey RP , et al. Endothelial to mesenchymal transition in cardiovascular disease: JACC state‐of‐the‐art review. J Am Coll Cardiol. 2019;73:190‐209. 30654892 10.1016/j.jacc.2018.09.089 PMC6865825

[57] Popov D . Endothelial cell dysfunction in hyperglycemia: Phenotypic change, intracellular signaling modification, ultrastructural alteration, and potential clinical outcomes. Inter J Diabetes Mellit. 2010;2:189‐195.

[58] Yu CH , Suriguga GM , Liu WJ , Cui NX , Wang Y . Du X and Yi ZC. High glucose induced endothelial to mesenchymal transition in human umbilical vein endothelial cell. Exp Mol Pathol. 2017;102:377‐383. 28347704 10.1016/j.yexmp.2017.03.007

[59] Yoshimatsu Y , Watabe T . Roles of TGF‐beta signals in endothelial‐mesenchymal transition during cardiac fibrosis. Int J Inflam. 2011;2011:724080. 22187661 10.4061/2011/724080 PMC3235483

[60] Mahler GJ , Farrar EJ , Butcher JT . Inflammatory cytokines promote mesenchymal transformation in embryonic and adult valve endothelial cells. Arterioscler Thromb Vasc Biol. 2013;33:121‐130. 23104848 10.1161/ATVBAHA.112.300504 PMC3694265

[61] Ma X , Zhao D , Yuan P , et al. Endothelial‐to‐mesenchymal transition in calcific aortic valve disease. Acta Cardiol Sin. 2020;36:183‐194. 32425433 10.6515/ACS.202005_36(3).20200213A PMC7220963

[62] Zeisberg EM , Potenta SE , Sugimoto H , Zeisberg M , Kalluri R . Fibroblasts in kidney fibrosis emerge via endothelial‐to‐mesenchymal transition. J Am Soc Nephrol. 2008;19:2282‐2287. 18987304 10.1681/ASN.2008050513 PMC2588112

[63] Yuan C , Ni L , Zhang C , Xia H , Wu X . Ephrin B2 mediates high glucose induced endothelial‐to‐mesenchymal transition in human aortic endothelial cells. Cardiovasc Diagn Ther. 2020;10:778‐785. 32968633 10.21037/cdt-20-299 PMC7487366

[64] Vadana M , Cecoltan S , Ciortan L , et al. Molecular mechanisms involved in high glucose‐induced valve calcification in a 3D valve model with human valvular cells. J Cell Mol Med. 2020;24:6350‐6361. 32307869 10.1111/jcmm.15277 PMC7294117

[65] Ciortan L , Macarie RD , Cecoltan S , et al. Chronic high glucose concentration induces inflammatory and remodeling changes in valvular endothelial cells and valvular interstitial cells in a gelatin methacrylate 3D model of the human aortic valve. Polymers (Basel). 2020;12(12):2786. 10.3390/polym12122786 PMC7760928 33255639

[66] Arevalos CA , Berg JM , Nguyen JM , Godfrey EL , Iriondo C , Grande‐Allen KJ . Valve interstitial cells act in a pericyte manner promoting angiogensis and invasion by valve endothelial cells. Ann Biomed Eng. 2016;44:2707‐2723. 26905695 10.1007/s10439-016-1567-9 PMC4983529

[67] Gendron N , Rosa M , Blandinieres A , et al. Human aortic valve interstitial cells display proangiogenic properties during calcific aortic valve disease. Arterioscler Thromb Vasc Biol. 2021;41:415‐429. 33147990 10.1161/ATVBAHA.120.314287

[68] Xu K , Xie S , Huang Y , et al. Cell‐type transcriptome atlas of human aortic valves reveal cell heterogeneity and endothelial to mesenchymal transition involved in calcific aortic valve disease. Arterioscler Thromb Vasc Biol. 2020;40:2910‐2921. 33086873 10.1161/ATVBAHA.120.314789

[69] Scatena M , Jackson MF , Speer MY , Leaf EM , Wallingford MC , Giachelli CM . Increased calcific aortic valve disease in response to a diabetogenic, procalcific diet in the LDLr(‐/‐)ApoB(100/100) mouse model. Cardiovasc Pathol. 2018;34:28‐37. 29539583 10.1016/j.carpath.2018.02.002 PMC5940574

[70] Seliga JOM , Raschkea S , Thoresene H , et al. Impact of hyperinsulinemia and hyperglycemia on valvular interstitial cells–a link between aortic heart valve degeneration and type 2 diabetes. BA‐Mol Basis Dis. 2019;1865:2526‐2537. 10.1016/j.bbadis.2019.05.019 31152868

[71] Bruderer M , Richards RG , Alini M , Stoddart MJ . Role and regulation of RUNX2 in osteogenesis. Eur Cell Mater. 2014;28:269‐286. 25340806 10.22203/ecm.v028a19

[72] Cheek JD , Wirrig EE , Alfieri CM , James JF , Yutzey KE . Differential activation of valvulogenic, chondrogenic, and osteogenic pathways in mouse models of myxomatous and calcific aortic valve disease. J Mol Cell Cardiol. 2012;52:689‐700. 22248532 10.1016/j.yjmcc.2011.12.013 PMC3294059

[73] Wirrig EEY KE . Developmental pathways in CAVD. Calcific Aortic Valve Disease; IntechOpen Limited: . 2013.

[74] Nagy E , Eriksson P , Yousry M , et al. Valvular osteoclasts in calcification and aortic valve stenosis severity. Int J Cardiol. 2013;168:2264‐2271. 23452891 10.1016/j.ijcard.2013.01.207

[75] Voicu G , Rebleanu D , Constantinescu CA , et al. Nano‐polyplexes mediated transfection of runx2‐shRNA mitigates the osteodifferentiation of human valvular interstitial cells. Pharmaceutics. 2020;12(6):507. 10.3390/pharmaceutics12060507 PMC7355966 32498305

[76] Filippi A , Constantin A , Alexandru N , et al. Integrins alpha4beta1 and alphaVbeta3 are reduced in endothelial progenitor cells from diabetic dyslipidemic mice and may represent new targets for therapy in aortic valve disease. Cell Transplant. 2020;29:963689720946277. 32841051 10.1177/0963689720946277 PMC7563030

[77] Abplanalp WT , Conklin DJ , Cantor JM , et al. Enhanced integrin alpha4beta1‐mediated adhesion contributes to a mobilization defect of endothelial progenitor cells in diabetes. Diabetes. 2016;65:3505‐3515. 27495221 10.2337/db16-0634 PMC5079633

[78] Colazzo F , Sarathchandra P , Smolenski RT , et al. Extracellular matrix production by adipose‐derived stem cells: implications for heart valve tissue engineering. Biomaterials. 2011;32:119‐127. 21074262 10.1016/j.biomaterials.2010.09.003

[79] Constantin A , Filippi A , Alexandru N , Nemecz M , Georgescu A . Extracellular vesicles from adipose tissue stem cells in diabetes and associated cardiovascular disease; pathobiological impact and therapeutic potential. Int J Mol Sci. 2020;21(24):9598. 10.3390/ijms21249598 PMC7766415 33339409

[80] Schoen FJ . Morphology, clinicopathologic correlations, and mechanisms in heart valve health and disease. Cardiovasc Eng Technol. 2018;9:126‐140. 27502286 10.1007/s13239-016-0277-7

[81] Frasca A , Xue Y , Kossar AP , et al. Glycation and serum albumin infiltration contribute to the structural degeneration of bioprosthetic heart valves. JACC Basic Transl Sci. 2020;5:755‐766. 32875167 10.1016/j.jacbts.2020.06.008 PMC7452200

[82] Toshima T , Watanabe T , Narumi T , et al. Therapeutic inhibition of microRNA‐34a ameliorates aortic valve calcification via modulation of Notch1‐Runx2 signaling. Cardiovasc Res. 2020;116(5):983‐994. 31393559 10.1093/cvr/cvz210

[83] Simionescu DT , Chen J , Jaeggli M , Wang B , Liao J . Form follows function: advances in trilayered structure replication for aortic heart valve tissue engineering. J Healthc Eng. 2012;3:179‐202. 23355946 10.1260/2040-2295.3.2.179 PMC3552623

[84] Chow JP , Simionescu DT , Warner H , et al. Mitigation of diabetes‐related complications in implanted collagen and elastin scaffolds using matrix‐binding polyphenol. Biomaterials. 2013;34:685‐695. 23103157 10.1016/j.biomaterials.2012.09.081 PMC3496025

[85] Bouchareb R , Guauque‐Olarte S , Snider J , et al. Proteomic architecture of valvular extracellular matrix: FNDC1 and MXRA5 are new biomarkers of aortic stenosis. JACC Basic Transl Sci. 2021;6:25‐39. 33532664 10.1016/j.jacbts.2020.11.008 PMC7838057

[86] Redfors B , Furer A , Lindman B , et al. Biomarkers in aortic stenosis: a systematic review. Structural Heart. 2017;1:18‐30.

[87] Nader J , Metzinger L , Maitrias P , Caus T , Metzinger‐Le MV . Aortic valve calcification in the era of non‐coding RNAs: the revolution to come in aortic stenosis management? Noncoding RNA Res. 2020;5(2):41‐47. 10.1016/j.ncrna.2020.02.005 32195449 PMC7075756

[88] Toshima T , Watanabe T , Narumi T , et al. Therapeutic inhibition of microRNA‐34a ameliorates aortic valve calcification via modulation of Notch1‐Runx2 signalling. Cardiovasc Res. 2020;116(5):983‐994. 10.1093/cvr/cvz210. PMID: 31393559. 31393559

[89] Harpa M , Branzaniuc K , Movileanu I , et al. Implantation of valvular collagen biomaterials seeded with autologous stem cells–intraoperative hemodynamic measurements in an animal model. Acta Medica Transilvanica. 2015;20:38‐39.

[90] Harpa M , Movileanu I , Sierad L , et al. In vivo testing of xenogeneic acellular aortic valves seeded with stem cells. Rev Rom Med Lab. 2016;24:343‐346. 31098341 10.1515/rrlm-2016-0031 PMC6516535

[91] Harpa MMMI , Sierad LN , Cotoi OS , et al. Simionescu D Pulmonary heart valve replacement using stabilized acellular xenogeneic scaffolds; effects of seeding with autologous stem cells. Rev Romana Med Lab. 2015;23:415‐429.

[92] Chow JP , Simionescu DT , Carter AL , Simionescu A . Immunomodulatory effects of adipose tissue‐derived stem cells on elastin scaffold remodeling in diabetes. Tissue Eng Regen Med. 2016;13:701‐712. 30603451 10.1007/s13770-016-0018-x PMC6170861

[93] Atkins SK , Aikawa E . Valve under the microscope: shining a light on emerging technologies elucidating disease mechanisms. Heart. 2019;105:1610‐1611. 31296591 10.1136/heartjnl-2019-315236 PMC6855786

[94] Greene CL , Jaatinen KJ , Wang H , Koyano TK , Bilbao MS , Woo YJ . Transcriptional profiling of normal, stenotic, and regurgitant human aortic valves. Genes (Basel). 2020;11(7):789. 10.3390/genes11070789 PMC7397246 32674273

[95] Krohn JB , Hutcheson JD , Martínez‐Martínez E , Aikawa E . Extracellular vesicles in cardiovascular calcification: expanding current paradigms. J Physiol. 2016;594(11):2895‐2903. 10.1113/JP271338. PMID: 26824781; PMCID: PMC4887674. 26824781 PMC4887674

[96] Zhang BL , Bianco RW , Schoen FJ . Preclinical assessment of cardiac valve substitutes: current status and considerations for engineered tissue heart valves. Front Cardiovasc Med. 2019;7(6):72. 10.3389/fcvm.2019.00072. PMID: 31231661; PMCID: PMC6566127. PMC6566127 31231661

[97] Scatena M , Jackson MF , Speer MY , Leaf EM , Wallingford MC , Giachelli CM . Increased calcific aortic valve disease in response to a diabetogenic, procalcific diet in the LDLr ‐/‐ ApoB 100/100 mouse model. Cardiovasc Pathol. 2018;34:28‐37. 10.1016/j.carpath.2018.02.002. Epub 2018 Feb 15. PMID: 29539583; PMCID: PMC5940574. 29539583 PMC5940574

[98] Zabirnyk A , Perez MDM , Blasco M , et al. A novel ex vivo model of aortic valve calcification. a preliminary report. Front Pharmacol. 2020;17(11):10.3389/fphar.2020.568764. PMID: 33390945; PMCID: PMC7773652. 568764. PMC7773652 33390945

[99] Lee MH , Arcidiacono JA , Bilek AM , et al. Considerations for tissue‐engineered and regenerative medicine product development prior to clinical trials in the United States. Tissue Eng Part B Rev. 2010;16(1):41‐54. 10.1089/ten.TEB.2009.0449. PMID: 19728784. 19728784

[100] Yutzey KE , Demer LL , Body SC , et al. Calcific aortic valve disease: a consensus summary from the Alliance of Investigators on Calcific Aortic Valve Disease. Arterioscler Thromb Vasc Biol. 2014;34(11):2387‐2393. 10.1161/ATVBAHA.114.302523. Epub 2014 Sep 4. PMID: 25189570; PMCID: PMC4199903. 25189570 PMC4199903

